# Development and validation of a minimally invasive diagnostic model for biliary atresia using artificial intelligence

**DOI:** 10.1007/s12519-025-00988-2

**Published:** 2025-11-11

**Authors:** Jing-Ying Jiang, Rui Dong, Ying-Hua Sun, Yi-Fan Yang, Henkjan J. Verkade, Xiao Cai, Xiao-Li Xie, Zhi-Bo Zhang, Zhong-Xi Zhang, Zhu Jin, Min Du, Jian-Jun Zhang, Zhen Shen, Wei-Li Yan, Gong Chen, Shan Zheng

**Affiliations:** 1 Department of Pediatric Surgery, Shanghai Key Laboratory of Birth Defect, and Key Laboratory of Neonatal Disease, Children’s Hospital of Fudan University, Ministry of Health, 399 Wan Yuan Rd, Shanghai 201102, China; 2 Department of Ultrasound, Children’s Hospital of Fudan University, Shanghai 201102, China; 3 Department of Pediatrics, University of Groningen, University Medical Center Groningen, Groningen, The Netherlands; 4 Department of Population Health Sciences, Weill Cornell Medicine, New York, 10065 USA; 5 Department of Pediatric Gastroenterology, School of Medicine, Chengdu Women’s and Children’s Central Hospital, University of Electronic Science and Technology of China, Chengdu 610091, China; 6 Department of Pediatric Surgery, Shengjing Hospital of Chinese Medical University, Shenyang 110004, China; 7 Department of Pediatric Surgery, Xiamen Children’s Hospital, Xiamen 361006, China; 8 Department of Pediatric Surgery, Affiliated Hospital of Zunyi Medical University, Zunyi 563000, China; 9 Department of Pediatric Surgery, Xuzhou Children’s Hospital, Xuzhou 221002, China; 10 Department of Clinical Epidemiology, Clinical Trial Unit (CTU), Children’s Hospital of Fudan University, 399 Wan Yuan Road, Shanghai 201102, China

**Keywords:** Artificial intelligence, Biliary atresia, Diagnostic model, Matrix metalloproteinase-7, Ultrasound

## Abstract

**Background:**

Ultrasound and serum matrix metalloproteinase-7 (MMP-7) hold great value in distinguishing biliary atresia (BA) from other cholestatic diseases. This study aims to assess the accuracy of an artificial intelligence (AI) based diagnostic model of ultrasound combined with serum MMP-7 in discriminative diagnosis of BA.

**Methods:**

This is a multicenter diagnostic study involving six medical centers in China. Patients with obstructive jaundice were enrolled. A set of morphological operators were employed to extract features of the ultrasound images to construct an AI algorithm. Logistic regression model was established with validation.

**Results:**

Two cohorts with a total of 348 children with obstructive jaundice were recruited from January 2020 to April 2023. A retrospective cohort of 187 infants served as a training cohort; this included 56 BA and 131 non-BA patients. Serum MMP-7 testing model yielded an area under the receiver-operating characteristic curve (AUROC) of 0.916 [95% confidence interval (CI) = 0.876–0.956], sensitivity of 94.6% (95% CI = 85.1%–98.9%), specificity of 88.6% (95% CI = 81.8%–93.5%), and accuracy of 90.4% (95% CI = 85.2%–94.2%). Values for ultrasound testing model were 0.945 (95% CI = 0.902–0.987), 98.2% (95% CI = 90.5%–99.9%), 91.6% (95% CI = 85.5%–95.7%), and 93.6% (95% CI = 89.1%–96.6%), respectively. The combined AI model obtained an AUROC of 0.985 (95% CI = 0.971–0.999), sensitivity of 98.2% (95% CI = 90.5%–99.9%), specificity of 93.1% (95% CI = 84.4%–96.4%), and accuracy of 94.7% (95% CI = 90.4%–97.4%), respectively. Performance was confirmed using a multicenter prospective validation cohort of 161 patients that included 100 BA cases.

**Conclusion:**

An AI model combining ultrasound and serum MMP-7 demonstrated robust high sensitivity and specificity in the differential diagnosis of BA.

**Graphical abstract:**

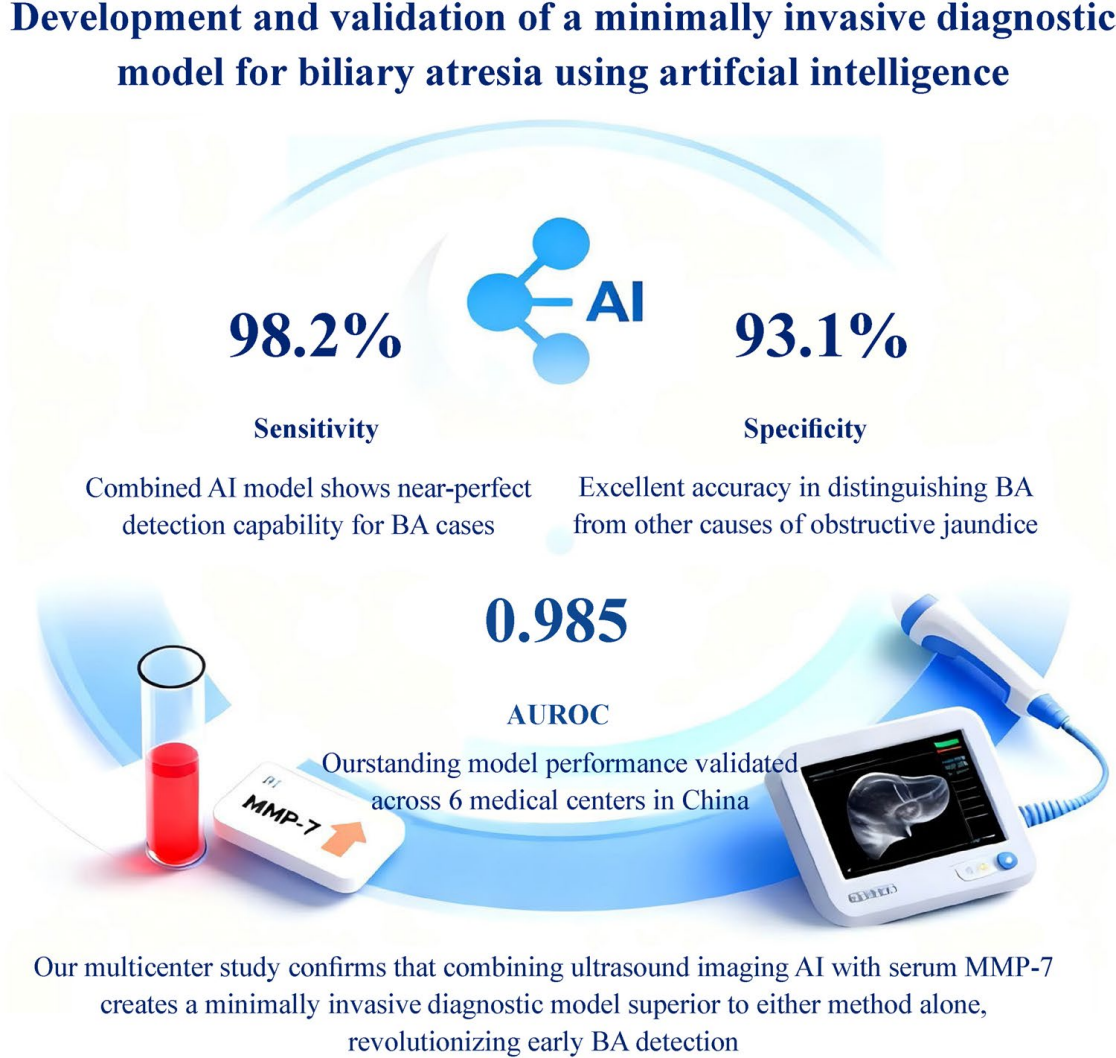

**Supplementary Information:**

The online version contains supplementary material available at 10.1007/s12519-025-00988-2.

## Introduction

Biliary atresia (BA) is a severe liver disease that affects 1/8000–10,000 neonates. BA is characterized by destruction of bile ducts and subsequent liver fibrosis [1]. Kasai portoenterostomy (KPE) has been the standard treatment strategy to restore bile flow. The outcome of KPE depends on patient age at surgery and those operated upon within 60 days after birth generally achieve better outcomes [2–5]. Intraoperative cholangiography, an invasive test, is the gold standard for diagnosing BA. Indeed, around 10% of cholestatic infants were excluded from a diagnosis of BA following intraoperative cholangiography [6]. Accurate and noninvasive methods to differentiate BA from other non-BA cholestatic diseases as early as possible are crucial to surgical decision-making; however, this is a challenge due to overlapping symptoms and test results.

Ultrasonography (US) has been widely used for initial evaluation of suspected BA. Multiple features are helpful in discriminating BA from other causes of cholestasis, achieving diagnostic accuracy of over 90% [7–9]. For example, the triangular cord sign is echogenic triangular fibrous tissue anterior to the right portal vein near the porta hepatis and represents obliterated biliary remnants [10–13]. Other ultrasonographic features include absence of or abnormal gallbladder morphology, enlarged hepatic artery, absence of common bile duct, and elevated shear wave elastography [14–16]. However, the accuracy of US largely relies on the experience of pediatric radiologists. Recent studies have attempted to use deep machine learning models in decision-making. However, most of these were based on US features manually extracted by sonographers [17–21] potentially limiting performance in broader real-world settings. In 2021, Zhou et al. established and validated an ensembled deep learning model for the diagnosis of BA based on sonographic gallbladder images that showed superior performance to human experts [20]. This model was based on the gallbladder alone which might be limiting where the gallbladder is absent or US is unable to localize the gallbladder region.

Matrix metalloproteinase-7 (MMP-7) testing, as an objective method, has been identified in numerous studies as a highly specific serum biomarker in the diagnosis of BA. This test consistently demonstrates superior discriminatory performance compared to the conventional liver function tests, with reported area under the receiver-operating characteristic (ROC) curves of over 0.9 [22–35]. Improved diagnostic accuracy of the combination of US and serum MMP-7 test was expected in the study of Pandurangi et al. [28].

In the present study, we aimed to explore the combination of subjective US and objective serum MMP-7 testing as a potential minimally invasive preoperative method to improve diagnostic accuracy of BA in a Chinese clinical setting.

## Methods

### Study design

This study was performed in compliance with the Declaration of Helsinki and approved by the Ethics Committee of Children’s Hospital of Fudan University (No. 2021-443). The study was prospectively registered at http://www.chictr.org.cn/ (CHiCTR2200055459). Informed consent was obtained from the guardian of each participant before enrollment.

This was a multicenter diagnostic accuracy study involving two cohorts of infant patients. The diagnostic performance of an AI model combining serum MMP-7 testing and image-based ultrasound algorithms was compared with the two tests separately in a single-center retrospective cohort. The model was then validated in a prospectively recruited multicenter cohort.

### Study setting and subjects

The training cohort was enrolled at the Children’s Hospital of Fudan University between January, 2020 and December, 2021 (*n* = 187). Infants aged below 150 days and diagnosed with BA or non-BA cholestasis according to medical records were enrolled. The validation cohort was prospectively and consecutively enrolled from the surgery department of six geographic urban tertiary care academic children’s hospitals in China between January, 2022 and March, 2023 [Children’s Hospital of Fudan University (*n* = 83), Chengdu Women’s and Children’s Central Hospital (*n* = 41), Shengjing Hospital of Chinese Medical University (*n* = 25), Xiamen Children’s Hospital (*n* = 4), Affiliated Hospital of Zunyi Medical University (*n* = 4), and Xuzhou Children’s Hospital (*n* = 4)]. In this cohort, infants aged below 150 days who met the criteria of cholestasis [serum total bilirubin (TB) ≥ 85 µmol/L, but direct bilirubin (DB) accounting for more than 20% of TB, or serum TB < 85 µmol/L, but serum DB ≥ 17 µmol/L] were eligible. Exclusion criteria included: (1) other severe congenital malformations or immunodeficiencies; (2) inability to complete all index tests prior to reference testing; and (3) refusal to take further consultation and treatment to obtain a definitive diagnosis.

### Diagnostic tests under investigation

#### Serum matrix metalloproteinase-7

Serum samples were obtained at admission and stored at – 80 ℃ before assay. Serum MMP-7 concentrations were measured using an enzyme-linked immunosorbent assay (ELISA) kit (R&D Systems, DMP700, Minneapolis, MN, USA), based on 20-fold diluted serum samples. All measurements were performed in a central study laboratory (Children’s Hospital of Fudan University) by WuXi Diagnostics (Shanghai, China), who were blinded to other test results. The precision of the MMP-7 ELISA was validated by WuXi Diagnostics; intra and inter-assay coefficients of variation (CV) were < 5 and < 10%, respectively, confirming high reproducibility. Each sample was assayed in triplicate with the mean used for analysis. Based on our previous research, a positive result was defined as > 18 ng/mL for infants > 30 days old and > 28.1 ng/mL for infants ≤ 30 days old [27].

#### Ultrasound examination and image preprocessing

For each patient, a detailed gray-scale ultrasound examination was performed by experienced sonography experts at each center. Ultrasound systems used across the six study sites included: Philips EPIQ 7 (Philips Healthcare, Amsterdam, The Netherlands), Siemens ACUSON Sequoia (Siemens Healthineers, Erlangen, Germany), and Mindray Resona 7 (Mindray Bio-Medical Electronics Co., Shenzhen, China). A variety of convex array transducers were used, with frequencies ranging from 2 to 5 MHz (for abdominal scanning), appropriate for infant size. The specific machine and transducer used for each examination were recorded. Features included for analysis were presence of the triangular cord sign, presence/absence of the gallbladder, presence/absence of the common bile duct, hepatic artery diameter, and gallbladder morphology. Images of patients’ gallbladder and possible area of the triangular cord sign were recorded. All images were reviewed by a senior sonography expert (SYH). If there was inconsistency between the original measurement and the senior sonographer, the images would be reuploaded or excluded from final analysis.

Image pixels were converted to matrices of gray-scale values (range from 0 to 255, 0 means black, 255 means white) for further analysis. To unify images from different ultrasound devices, image normalization was performed to produce the same pixel intensity levels. Mean of all pixels in each image was set to 55 and interquartile range was set to 40. Normalized images were used for further analysis.

To focus on the region of interest (ROI), the ultrasound images were manually cropped to exclude extensive irrelevant areas. A series of morphological operations were applied to delineate the boundaries of key anatomical structures within the ROI, such as the gallbladder and portal vein. Using the extracted edges, a binary mask was generated to isolate the ROI. Statistical features, including the mean, median, and standard deviation of pixel intensities, were subsequently calculated for both the masked ROI and the entire cropped image.

Three categories of features were derived from the analysis: gray-scale characteristics, size attributes, and texture properties of the ROI. Grayscale features were obtained by analyzing quantile values from both the ROI and the entire cropped image. Size-related features included the absolute area of the ROI as well as its relative area ratio to the cropped image. Texture features were quantified using the standard deviation of pixel intensities within the ROI and the full cropped region. All extracted features were subsequently used for further analysis (Supplementary Fig. 1).

#### Image-based ultrasound artificial intelligence algorithm

Logistic regression was applied to establish the AI diagnostic model. All subjects with at least one ROI visible in the ultrasound images were included for analysis. Numerical variables were scaled to 0–1. Lasso was used to select variables to reduce the complexity of the model and avoid overfitting in the training cohort (*n* = 187). The selected features offer clinicians clear and interpretable prediction principles, facilitating a deeper understanding of the decision-making process of the model. The model was further validated using a multicenter prospective cohort involving six participating centers (*n* = 161).

#### Reference test

After assessing patient demographic characteristics and medical histories, biochemical testing was performed in all patients. This included TB, DB, gamma-glutamyl transferase (GGT), aspartate aminotransferase (AST), alanine aminotransferase (ALT), and total bile acid (TBA). In the prospective validation cohort, the results of the serum MMP-7 and ultrasound were integrated into the clinical workflow. The decision to proceed to intraoperative cholangiography was made by a pediatric surgeon based on a comprehensive assessment that included the results of these tests, alongside traditional indicators, such as acholic stool and abnormal biochemistry (e.g., high GGT). A positive MMP-7 result and/or positive AI ultrasound analysis significantly increased the clinical suspicion for BA and were key factors in the decision to recommend surgery. Otherwise, cholestatic infants would be closely followed up until the suspicion of BA was eliminated. BA diagnoses were made according to intraoperative cholangiography and subsequent histological examination of liver biopsies [1]. A non-BA diagnosis was made for intraoperative cholangiograms showing a patent biliary tree, percutaneous transhepatic biopsy excluding BA, genetic tests showing certain genetic mutations for cholestasis such as JAG 1 for Alagille syndrome, or alleviation of symptoms without surgical intervention by KPE during follow-up for at least 3 months. Unlike the retrospective training cohort, all diagnostic tests were performed before the reference tests.

### Statistical analysis

Demographic and clinical characteristics were summarized using conventional descriptive statistics: *n* (%) for categorical variables and median and quartiles (Q1, Q3) for continuous variables. Between-group comparisons were performed using the Chi-squared and Wilcoxon rank sum tests. A statistically significant difference was defined as a *P* value < 0.05.

ROC curves were constructed to assess the diagnostic ability of all tests under investigation. The area under the curve (AUC) and its 95% confidence intervals (CIs) were presented as overall measures of accuracy; Delong test was performed for comparisons between index tests. Sensitivity, specificity, and accuracy and 95% CIs were reported as diagnostic performance for all tests in both training and validation cohorts. To further investigate whether the performance differs in the center that generated the AI ultrasound model (internal cohort) from the other centers (external cohort), a subgroup analysis was performed. Calibration curves were generated to assess agreement between predicted probabilities and observed outcomes for the serum MMP-7 test, the ultrasound AI model, and the combined AI model in both the training and validation cohorts.

No missing data were imputed and unadjusted *P* values were reported. Data analyses were performed using both R software 4.0.3 (R Foundation, Vienna, Austria) and Python software 3.1.0 (Python software Foundation, https://www.python.org).

## Results

### Study population

**Fig. 1:**
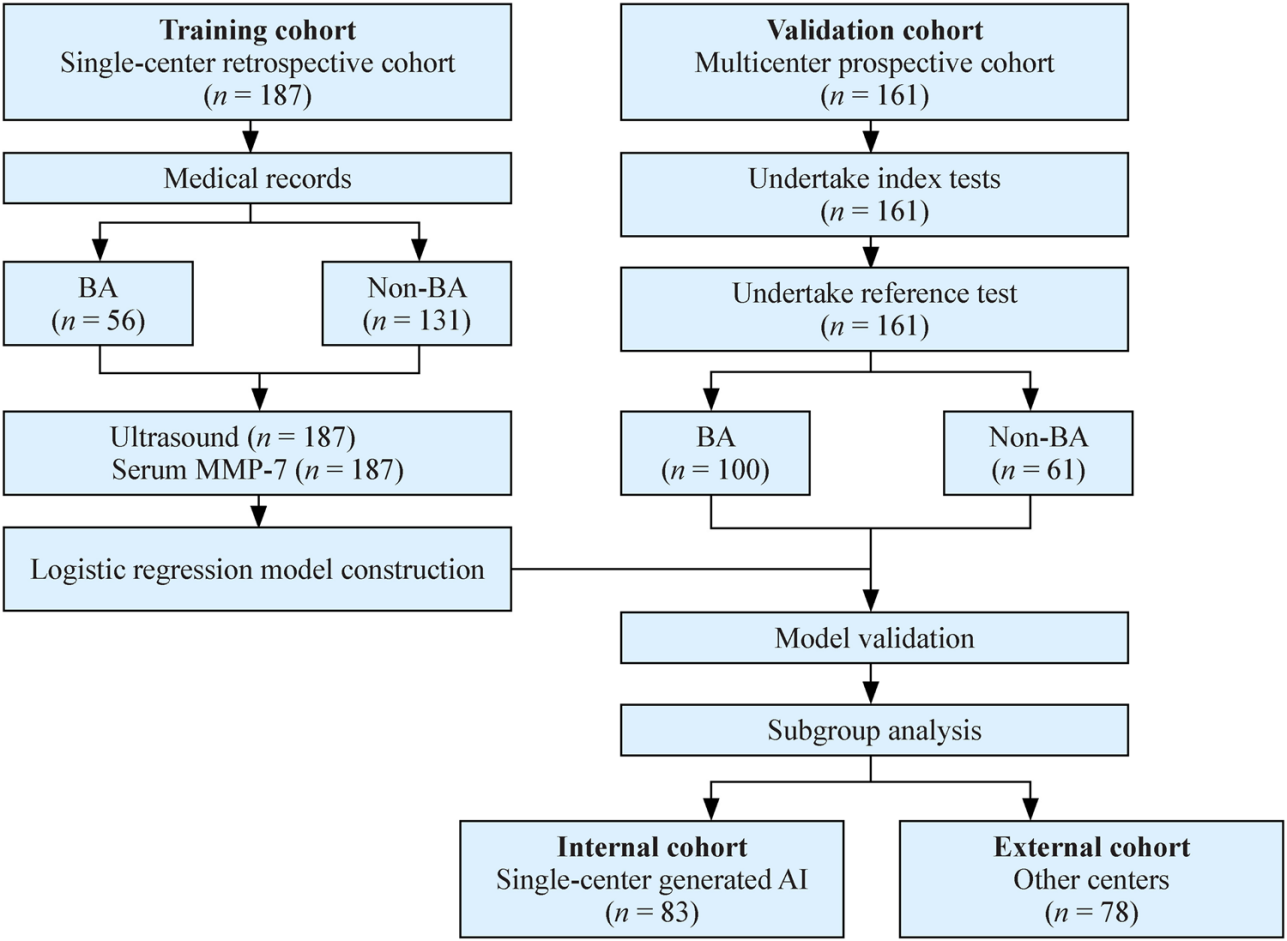
Study design, flowchart, and cohort makeup. *BA* biliary atresia, *MMP-7* matrix metalloproteinase-7, *AI* artificial intelligence

As shown in Fig. 1, 187 eligible infants from a single center were retrospectively enrolled as the training cohort, including 56 (29.9%) BA and 131 non-BA patients. In total, 161 infants were prospectively recruited from six medical centers as the validation cohort, including 100 (62.1%) BA and 61 non-BA (Table 1). Median levels of TB, DB, AST, ALT, and TBA were similar between the two cohorts, while the median GGT levels were higher and median age were lower by 4 days, which are probably related to the greater proportion of BA cases in the validation cohorts (Table 1). In both cohorts, BA cases were younger, with more females, higher levels of GGT, TB, and DB, as well as serum MMP-7 concentrations (Supplementary Table 1).

**Table 1:** Demographic and clinical characteristics of study cohorts

Variables	Training cohort (*n* = 187)	Validation cohort (*n* = 161)	*P*^a^
Sex (male)	101 (62.3)	88 (60.3)	0.798
BA	56 (29.9)	100 (62.1)	< 0.001
Age (d)	68.0 (51.0, 91.2)	64.0 (44.5, 79.0)	< 0.001
GGT (IU/L)	158.1 (87.0, 337.9)	281.7 (137.8, 639.5)	< 0.001
TB (µmol/L)	143.9 (108.6, 190.5)	145.2 (107.8, 175.9)	0.912
DB (µmol/L)	105.3 (80.2, 141.0)	98.7 (71.2, 127.8)	0.054
TBA (µmol/L)	98.1 (74.4, 121.3)	101.0 (75.1, 125.6)	0.426
ALT (IU/L)	134.5 (70.7, 213.7)	121.4 (69.1, 211.1)	0.399
AST (IU/L)	186.0 (119.0, 292.2)	178.0 (94.4, 285.4)	0.343

Data are presented as *n* (%) or median (interquartile range). *BA* biliary atresia, *GGT* gamma-glutamyl transferase, *TB* total bilirubin, *DB* direct bilirubin, *TBA* total bile acid, *ALT* alanine aminotransferase, *AST* aspartate aminotransferase. ^a^Pearson’s Chi-squared test was applied to sex and BA proportions. Wilcoxon rank sum test was applied to other parameters

### Image-based ultrasound artificial intelligence algorithm

Positive ultrasound features, including the presence of the triangular cord sign, presence/absence of the gallbladder, gallbladder morphology, presence/absence of the common bile duct, and hepatic artery diameter, were significantly more common in BA cases than in non-BA patients in the two cohorts (Supplementary Table 1). Model evaluation showed that three ultrasound images were valuable in model construction, including one for the gallbladder and two different sections for the triangular cord sign (the left and right branches of the portal vein and the transverse section of the right portal vein). After manually cropping ROIs, pixel normalization, and automatic extraction of features on ROIs, the image-based ultrasound AI algorithm was established.

### Diagnostic performance

The performance of a single ultrasound feature is presented in Supplementary Table 2 and Supplementary Fig. 2. This indicates that the triangular cord sign achieved the best diagnostic accuracy. The image-based ultrasound AI algorithm produced an AUC of 0.945 (95% CI = 0.902–0.987) in the training cohort, 0.909 (95% CI = 0.850–0.968) in the validation cohort, and the diagnostic accuracy was 93.6% (95% CI = 89.1%–96.6%) and 91.0% (95% CI = 85.4%–95.0%), respectively (Supplementary Table 2).

Serum MMP-7 produced an AUC of 0.916 (95% CI = 0.876−0.956) in the training cohort and 0.907 (95% CI = 0.857−0.957) in the validation cohort. The diagnostic accuracy was 90.4% (95% CI = 85.2%–94.2%) and 92.3% (95% CI = 87.0%–96.0%), respectively (Supplementary Table 2).

**Fig. 2:**
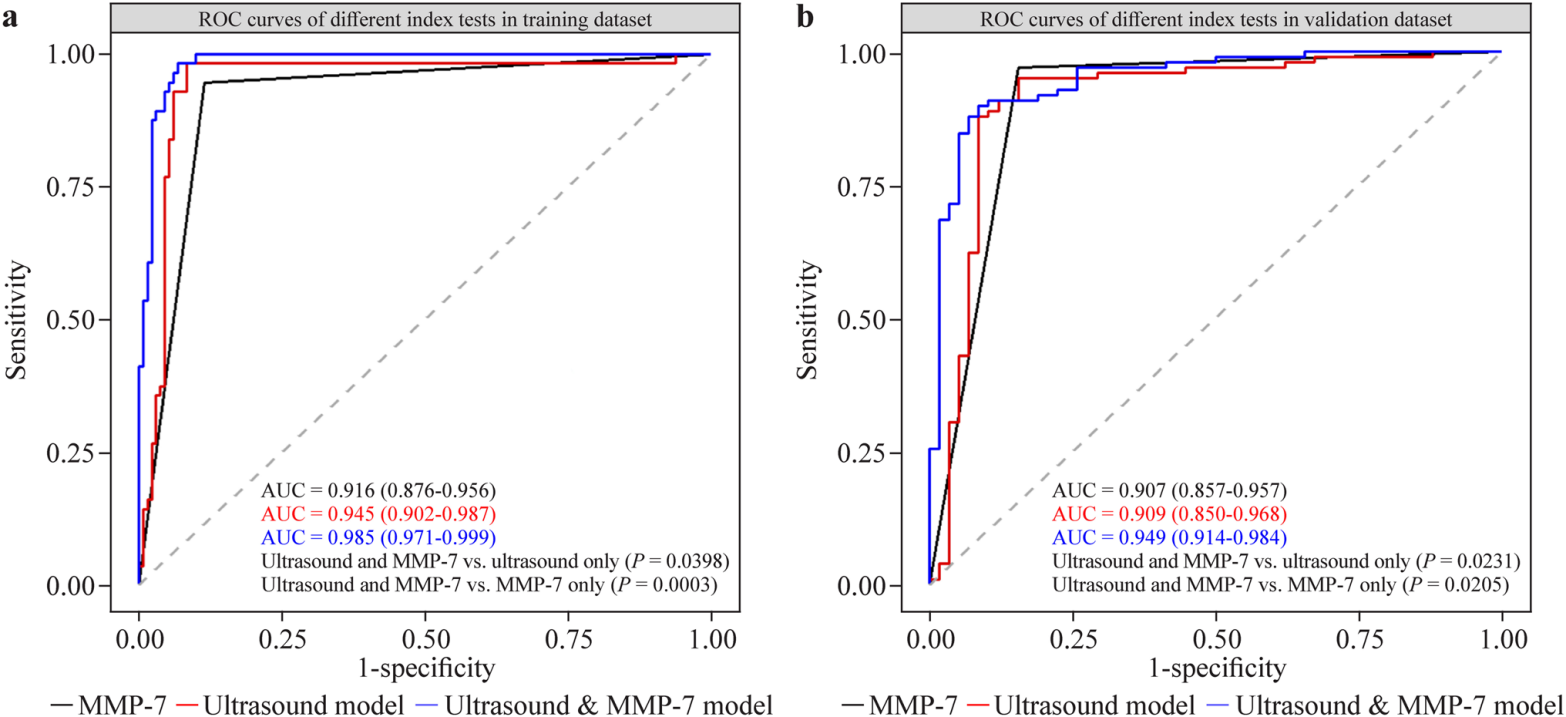
Diagnostic performance of different index tests. **a** ROC plots of artificial intelligence-based diagnostic models; serum MMP-7 alone, and ultrasound model alone based on the training cohort; **b** ROC plots of artificial intelligence-based diagnostic models; serum MMP-7 alone and ultrasound model alone based on the validation cohort. *ROC* receiver-operating characteristic, *AUC* area under the curve, *MMP-7* matrix metalloproteinase-7

The combined AI model using serum MMP-7 testing and image-based ultrasound significantly improved the AI algorithm with an AUC of 0.985 (95% CI = 0.971–0.999) in the training cohort; this was better than serum MMP-7 alone (AUC = 0.916, *P* = 0.0003) and ultrasound alone (AUC = 0.945, *P* = 0.04) (Fig. 2a). Sensitivity, specificity, and accuracy were 98.2% (95% CI = 90.5%–99.9%), 93.1% (95% CI = 84.4%–96.4%), and 94.7% (95% CI = 90.4%–97.4%), respectively. Performance of the combined method remained in the validation cohort with an AUC of 0.949 (95% CI = 0.914–0.984); sensitivity, specificity, and accuracy were 89.8% (95% CI = 82.0%–95.0%), 91.4% (95% CI = 81.0%–97.1%), and 90.4% (95% CI = 84.6%–94.5%), respectively (Table 2, Fig. 2b).

**Table 2:** Performance of combined AI diagnostic model of ultrasound and serum MMP-7

Cohort	AUC	Sensitivity, %	Specificity, %	Accuracy
Training	0.985 (0.971–0.999)	98.2 (90.5–99.9)	93.1 (84.4–96.4)	94.7 (90.4–97.4)
Validation	0.949 (0.914–0.984)	89.8 (82.0–95.0)	91.4 (81.0–97.1)	90.4 (84.6–94.5)
Internal	0.974 (0.944–1.000)	84.8 (73.0–92.8)	95.8 (78.9–99.9)	88.0 (79.0–94.2)
External	0.929 (0.869–0.989)	82.1 (66.5–92.5)	94.1 (80.3–99.3)	87.7 (77.9–94.2)

Data are presented as value (95% confidence interval). *AI* artificial intelligence, *MMP-7* matrix metalloproteinase-7, *AUC* area under the curve

**Fig. 3:**
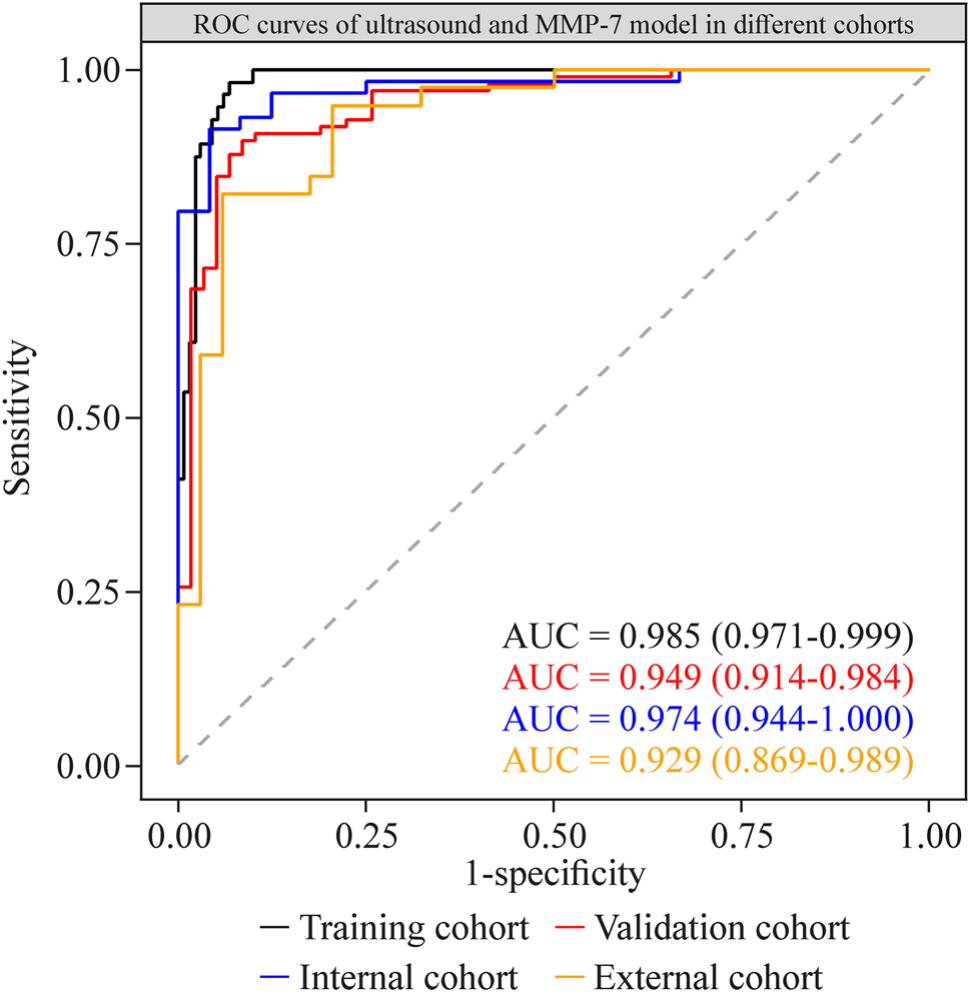
Diagnostic performance of an artificial intelligence-based diagnostic model using ultrasound and MMP-7; ROC plots for different cohorts. *ROC* receiver-operating characteristic, *AUC* area under the curve, *MMP-7* matrix metalloproteinase-7

Importantly, subgroup analysis showed that the sensitivity, specificity, and accuracy of the established model were comparable between the center that generated the AI ultrasound model (internal cohort) and the other centers (external cohort) (Table 2, Fig. 3).

The calibration curve for serum MMP-7 testing showed excellent agreement between predicted probabilities and observed outcomes. Conversely, for the validation cohort, the ultrasound AI model exhibited significant miscalibration. In contrast the combined AI model demonstrated strong overall calibration closely following the ideal trajectory, despite minor deviations in higher probability ranges (Supplementary Fig. 3).

### Development of AI tool

To facilitate clinical application of our AI-based diagnostic model, we developed a mobile application. To ensure functionality and accuracy, the program requires at least one US image including the gallbladder, or regions of the left and right branches of the portal vein or the transverse section of the right portal vein. In addition to the US images, input of serum MMP-7 value is mandatory. 

Upon opening the APP, the user is directed to crop the provided ultrasound image to adequately fit the ROI. Next, users are prompted to enter patient name, MMP-7 value, and age. Once the submission is confirmed, the APP processes the inputted data and the subsequent page displays a prediction; this indicates the likelihood of the patient being BA or non-BA. The user interface of the APP, which guides users through the submission and prediction process, is illustrated in Supplementary Fig. 4.

## Discussion

In this study we established an AI diagnostic model combining ultrasound and serum MMP-7 testing, yielding improved performance in differentiating BA from patients with similar symptoms. The model achieved 0.985 for AUROC, and sensitivity, specificity, and accuracy values of 98.2%, 93.1%, and 94.7%, respectively. These results were successfully validated in an independent prospective cohort. In addition, we developed our AI diagnostic model for use as a mobile application. This is expected to significantly enhance clinical applicability and improve minimally invasive preoperative diagnostic accessibility for BA. We hope that this model/APP will aid earlier BA diagnosis and subsequent Kasai surgery, crucial for achieving better long-term prognosis.

Accurate, early diagnosis and prompt surgery are critical in the management of BA. Serum biomarkers and ultrasound are two main tests in the diagnostic algorithm of BA. The selection of MMP-7 for this study was based on its superior diagnostic specificity for BA compared to standard liver function tests. In this study, the diagnostic accuracy of MMP-7 alone remained over 90% and was consistent with the previous studies [23–34]. The role of MMP-7 in the pathophysiology of BA remains unclear. Its diagnostic value may be largely due to an important role in remodeling the extracellular matrix, which is closely related to liver damage and progression to liver fibrosis [22, 36, 37], especially in BA patients [27, 36]. However, the false-negative rate of MMP-7 testing varied from 5% to 10%, leaving the chance for missed or delayed surgeries for true BA patients. For example, as hypothesized in our previous study [38], patients with false-negative MMP-7 test might be listed as a new clinical subtype of BA, showing pronounced damage in hepatic cells and cholangiocytes and impaired liver function leading to remodeling. Despite this, serum MMP-7 is regarded as an indirect but objective parameter in discriminating BA from other non-BA cholestasis.

Unlike serum MMP-7, ultrasound served as a direct but subjective diagnostic method, with the features of triangular cord sign and irregular or absent gallbladder being most suggestive for BA. In this study, we evaluated multiple sonographic features and confirmed that the triangular cord sign exhibited the highest diagnostic accuracy among the typical features assessed. However, it is important to note that a definitive triangular cord sign often reflects more advanced disease. To mitigate the influence of sonographer experience, patient age, and ultrasound device variability on diagnostic accuracy [39], we incorporated these factors into the development of our image-based AI algorithm. Our model achieved diagnostic performance comparable to that of a recently published ensemble deep learning model relying solely on gallbladder images [20]. In contrast to the approach of Zhou et al., our algorithm integrates features from the triangular cord region, enabling reliable analysis even in cases where the gallbladder is not visible. The significant miscalibration as shown on the calibration curve of the ultrasound-alone model in the validation cohort may be attributable in part to the multicenter nature of the study. Variations in ultrasound equipment and image quality across different centers, particularly between advanced and older devices, could introduce heterogeneity that challenges the consistency of image-based feature extraction and prediction.

To our knowledge, our combination model has not extensively been explored to date. We used features extracted from US images to match how human experts assess abnormalities in the gallbladder and portal hepatis (where triangular cord sign is located). Our diagnostic model offered superior performance as showed by prospective validation. Moreover, the consistent performance of our model in both internal and external cohorts (as shown by an external validation AUC of 0.929) as well as the good calibration of the combined model even in a high-prevalence setting further supports the reliability of the model and its effectiveness in diverse clinical settings.

In terms of clinical applicability, our study extends beyond academic inquiry through the implementation of our diagnostic algorithm within a user-friendly mobile application. Such mobile health tools have demonstrated considerable potential for enhancing clinical decision-making and healthcare delivery. By deploying this application, we aim to increase access to accurate BA diagnostics. The APP incorporates our integrated model, which requires both ultrasound images and MMP-7 values to generate a diagnosis. By combining objective biomarker measurement with AI-enhanced image analysis, the APP helps overcome diagnostic delays often associated with operator-dependent ultrasound interpretation or referral barriers. It provides clinicians with a rapid, evidence-based risk assessment, facilitating earlier referral for definitive surgical evaluation when needed, ultimately aiming to improve long-term outcomes for infants with BA.

There are several limitations to this study. First, our study included 348 cases, a substantial sample for a rare disease like BA, where large datasets are challenging to assemble. However, the prevalence of BA differed between the training cohort (29.9%) and the validation cohort (62.1%), primarily because the prospective validation cases were predominantly recruited from surgical departments, where disease suspicion was high. Despite this variation, the combined model demonstrated robust performance, with strong discrimination (AUC = 0.949), consistent accuracy of around 90% and excellent calibration alignment in the validation set. These results highlight the model’s reliability and generalizability across diverse prevalence scenarios and support its potential clinical utility in both primary screening and specialized diagnostic settings. Second, all images for analysis went through a process of manual cropping. This process may inevitably introduce bias and requires effort from the clinician to locate the ROI. Using popular models like U-net to realize complete automatically location of ROIs is an important direction of future research [40]. Training is necessary to standardize the process of ultrasound image selection and manual cropping of ROI before applying this AI diagnostic model. Nevertheless, the implementation of our combined AI model in low-resource or non-tertiary settings faces practical challenges. The dependence on serum MMP-7, a specialized biomarker not yet widely available, may restrict accessibility and the current need for manual image cropping requires operational expertise. Despite these limitations, our mobile application offers a viable pathway toward disseminating this diagnostic tool. Efforts are underway to promote standardized MMP-7 assay availability and further automate image processing. We anticipate that both the biomarker test and the AI model will become increasingly accessible across China and globally in the near future.

In conclusion, our study introduces a groundbreaking approach for the diagnosis of BA, combining ultrasound images with MMP-7. This novel combination model not only provides high diagnostic accuracy but also addresses challenges inherent in traditional diagnostic methods. The improved specificity, although not large, is of clinical significance to BA patients to avoid unnecessary diagnostic surgery. Our findings hold significant promise for enhancing early detection and accurate diagnosis of BA, potentially leading to improving patient outcomes.

## Supplementary Information

Below is the link to the electronic supplementary material.

Supplementary file 1 (DOCX 5275 KB)

## Data Availability

The data that support the findings of this study are available from the corresponding author upon reasonable request.
