# Supplementary material for: Development and validation of a minimally invasive diagnostic model for biliary atresia using artificial intelligence
Source: World J Pediatr. 2025 Nov 11;21(12):1289–98. doi: 10.1007/s12519-025-00988-2 (PMC12678471; doi:10.1007/s12519-025-00988-2)
Supplement: Supplementary file 1 — (DOCX 5275 KB) [file 12519_2025_988_MOESM1_ESM.docx]

**Supplementary Table 1**. Demographic and clinical characteristics of patients with biliary atresia and and non-biliary atresia in the three study cohorts

|  | **Training cohort** | | | **Validation cohort** | | |
| --- | --- | --- | --- | --- | --- | --- |
| **Variables** | **Non-BA^a^** | **BA^a^** | ***P*** | **Non-BA^a^** | **BA^a^** | ***P*** |
| *n* | 131 | 56 | < 0.001 | 61 | 100 | < 0.001 |
| Sex (male), *n* (%)b | 73 (68.9) | 28 (50.0) | 0.029 | 44 (73.3) | 44 (51.2) | 0.012 |
| Age (d)c | 73 (58, 93) | 55.5 (46.8, 71.8) | 0.004 | 67 (48, 86) | 51.5 (34.8, 67.2) | 0.002 |
| GGT (IU/L)c | 110.1 (67.1, 189.2) | 331.8 (178.5, 755.1) | < 0.001 | 134.7 (66, 230.9) | 492.8 (274.2, 802.5) | < 0.001 |
| TB (µmol/L)c | 133.5 (85.7, 189.7) | 157.5 (135.9, 192.3) | 0.003 | 128 (77.7, 169.9) | 150.1 (126.4, 183.4) | 0.003 |
| DB (µmol/L)c | 96.7 (68.9, 136.8) | 123.9 (102.5, 141.1) | < 0.001 | 82.5 (54, 125.1) | 106 (85, 128) | 0.003 |
| TBA (µmol/L)c | 93.6 (60.6, 121.3) | 105.4 (86.2, 121) | 0.06 | 100.7 (74.2, 134.1) | 102 (77.9, 124.3) | 0.984 |
| ALT (IU/L)c | 135 (66.9, 256.4) | 132.7 (75.8, 195.9) | 0.752 | 138.6 (69.5, 240.1) | 121.2 (68.9, 189.6) | 0.554 |
| AST (IU/L)c | 171.2 (104.1, 311.7) | 192 (132, 257.5) | 0.503 | 178 (86.2, 303) | 176.8 (105.8, 276) | 0.926 |
| Triangular cord sign, *n* (%)b | 2 (14.3) | 52 (100.0) | < 0.001 | 3 (5.5) | 76 (87.4) | < 0.001 |
| Abnormal gallbladder, *n* (%)b | 3 (21.4) | 49 (94.2) | < 0.001 | 15 (25.4) | 73 (83.0) | < 0.001 |
| Non-visualized CBD, *n* (%)b | 3 (21.4) | 52 (100.0) | < 0.001 | 32 (55.2) | 68 (77.3) | 0.009 |
| Enlarged hepatic artery, *n* (%)b | 6 (42.9) | 46 (88.5) | < 0.001 | 9 (16.1) | 47 (58.8) | < 0.001 |
| Serum MMP-7 (ng/mL)c | 10 (7.4, 13.4) | 50 (32.5, 96.4) | < 0.001 | 11.6 (8.1, 19.7) | 52.3 (33.9, 77.9) | < 0.001 |

*BA* biliary atresia, *GGT* gamma-glutamyl transferase, *TB* total bilirubin, *DB* direct bilirubin, *TBA* total bile acid, *ALT* alanine aminotransferase, *AST* aspartate aminotransferase, *MMP-7* matrix metalloproteinase-7. ^a^Median (interquartile range); ^b^Pearson's Chi-squared test; ^c^Wilcoxon rank sum test

**Supplementary Table 2**. Diagnostic performance of ultrasound and MMP-7

| **Variables** | **AUC** | **Sensitivity, %** | **Specificity, %** | **Accuracy** |
| --- | --- | --- | --- | --- |
| Triangular cord sign  Training cohort  Validation cohort | 0.929 (0.834-1.000)  0.910 (0.861-0.956) | 100.0 (93.2-100.0)  87.4 (78.5-93.5) | 85.7 (57.2-98.2)  94.6 (84.9-98.9) | 97.0 (89.5-99.6)  90.1 (84.0-94.5) |
| Abnormal gallbladder  Training cohort  Validation cohort | 0.896 (0.798-0.995)  0.863 (0.764-0.962) | 94.2 (84.1-98.8)  88.9 (70.8-97.7) | 78.6 (49.2-95.3)  75.8 (57.7-88.9) | 90.9 (81.3-96.6)  81.7 (69.6-90.5) |
| Non-visualized CBD  Training cohort  Validation cohort | 0.893 (0.781-1.000)  0.611 (0.532-0.689) | 100.0 (93.2-100.0)  77.3 (67.1-85.5) | 78.6 (49.2-95.3)  44.8 (31.7-58.5) | 95.5 (87.3-99.1)  64.4 (56.0-72.1) |
| Enlarged hepatic artery  Training cohort  Validation cohort | 0.728 (0.587-0.870)  0.814 (0.742-0.886) | 88.5 (76.6-95.7)  76.3 (65.4-85.1) | 57.1 (28.9-82.3)  75.0 (61.6-85.6) | 81.8 (70.4-90.2)  75.7 (67.6-82.7) |
| Ultrasound model  Training cohort  Validation cohort | 0.945 (0.902-0.987)  0.909 (0.850-0.968) | 98.2 (90.5-99.9)  94.9 (88.5-98.3) | 91.6 (85.5-95.7)  84.5 (72.6-92.7) | 93.6 (89.1-96.6)  91.0 (85.4-95.0) |
| Serum MMP-7  Training cohort  Validation cohort | 0.916 (0.876-0.956)  0.907 (0.857-0.957) | 94.6 (85.1-98.9)  96.9 (91.3-99.4) | 88.5 (81.8-93.4)  84.5 (72.6-92.7) | 90.4 (85.2-94.2)  92.3 (86.9-96.0) |

*MMP-7* matrix metalloproteinase-7, *AUC* area under the curve


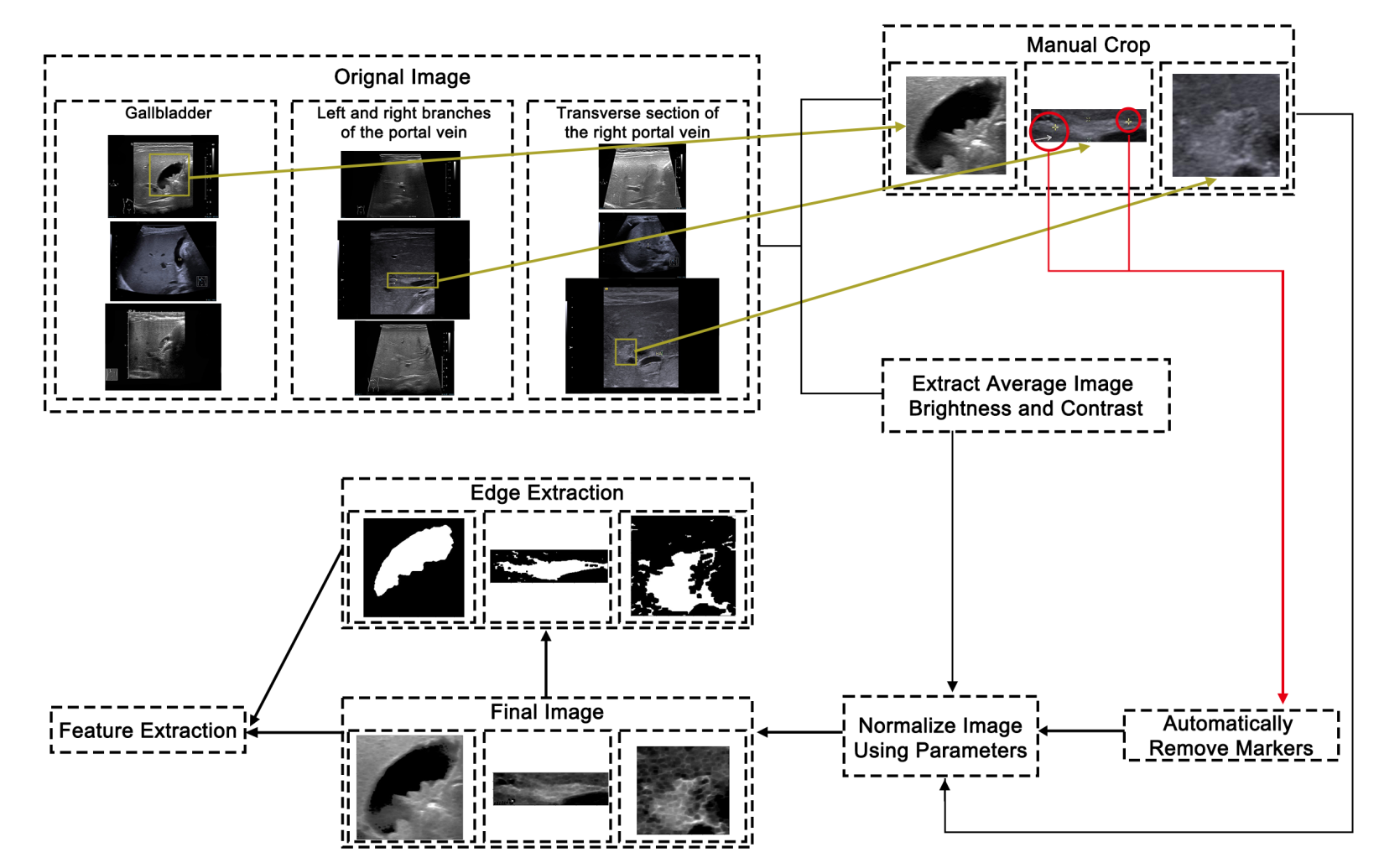


**Supplementary** **Fig. 1** Ultrasound image analysis process. After manually cropping region of interest (ROI) and normalizing pixels, the features on ROIs would be automatically extracted, including characteristics of grayscale, size and texture of the ROI


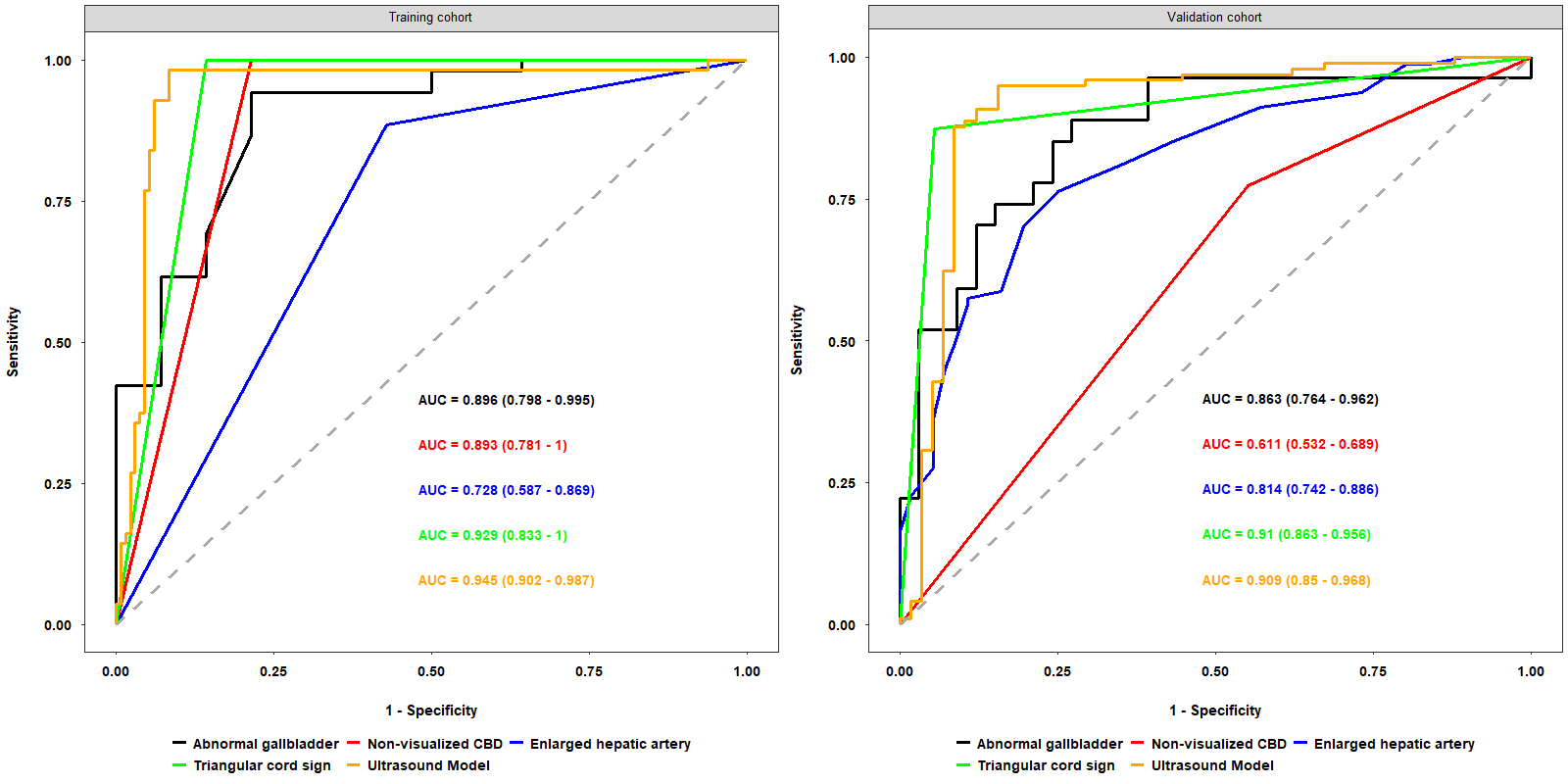


**Supplementary** **Fig. 2** Diagnostic accuracy of ultrasound features and model. **a** ROC plots based on the training cohort with AUC (95%CI); **b** ROC plots based on the validation cohort with AUC (95%CI). *ROC* receiver operating characteristic, *AUC* area under the curve, *CI* confidence interval


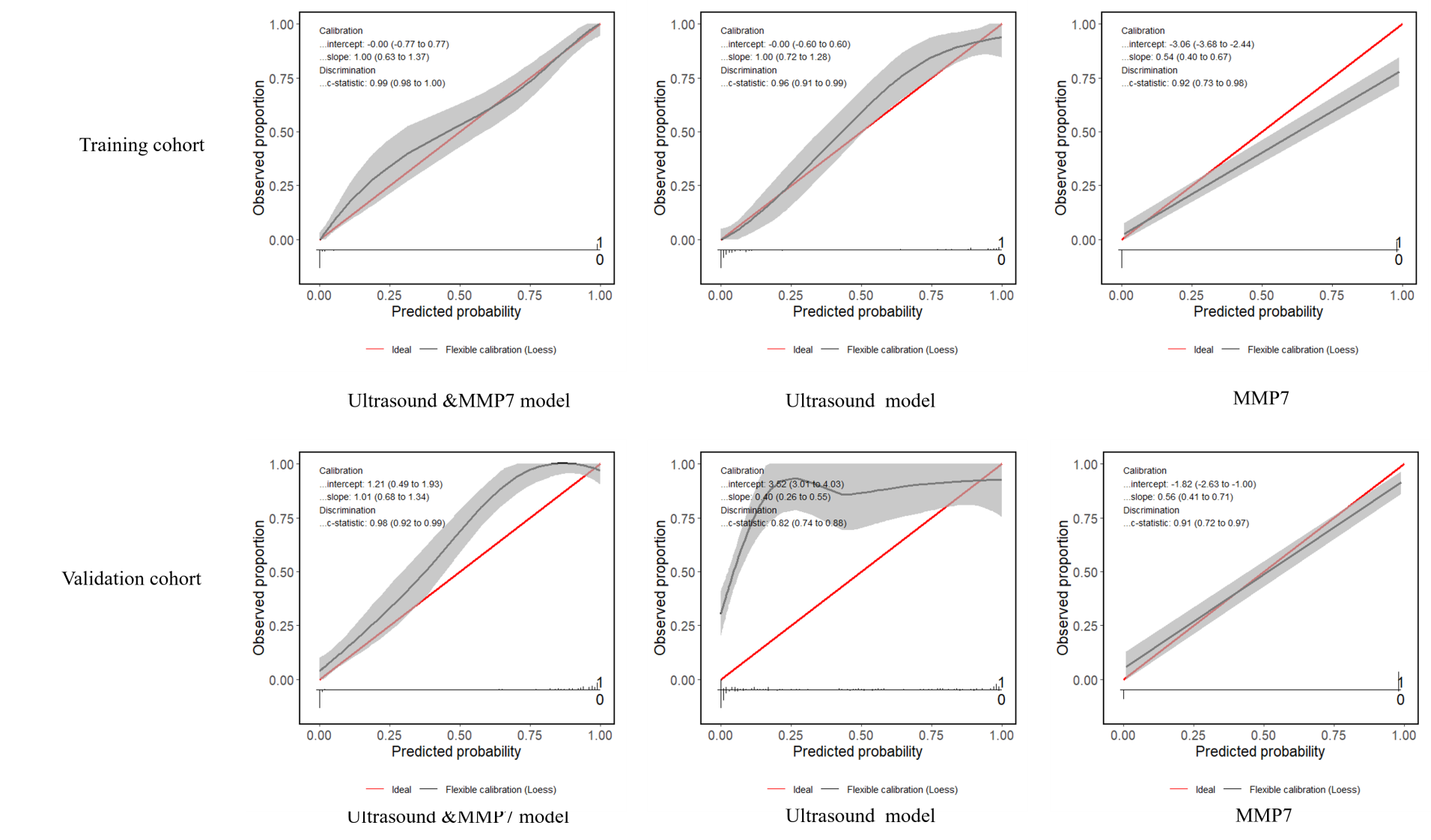


**Supplementary** **Fig. 3** Calibration curves for the serum MMP-7 test, ultrasound AI model and combined AI model in the training and validation cohorts. *MMP-7* matrix metalloproteinase-7, *AI* artificial intelligence


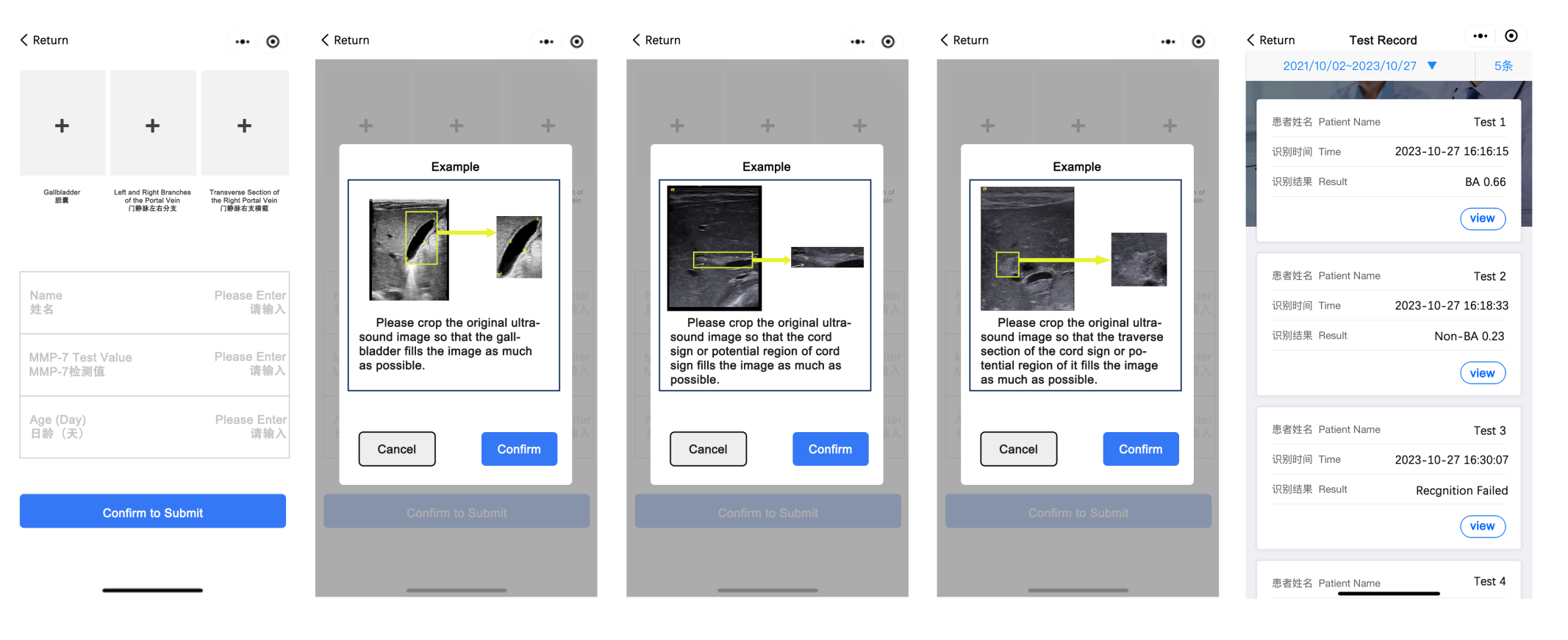


**Supplementary** **Fig. 4** Application interface of diagnostic model. By inputting the US images and serum MMP-7 value, the system will process the inputted data and display a prediction, indicating the likelihood of the patient being BA or not. *US* ultrasonography, *MMP-7* matrix metalloproteinase-7, *BA* biliary atresia
